# Hypoxia Alters Polymeric Immunoglobulin Receptor Expression and Cell Differentiation in the Airway Epithelium

**DOI:** 10.3390/ijms27219525

**Published:** 2026-10-24

**Authors:** Ludovic Gerard, Marylene Lecocq, Bruno Detry, Caroline Bouzin, Delphine Hoton, François Carlier, Thomas Plante-Bordeneuve, Sophie Gohy, Valérie Lacroix, Charles Pilette

**Affiliations:** 1 Department of Critical Care Medicine, Cliniques Universitaires Saint Luc, Université Catholique de Louvain (UCLouvain), Avenue Hippocrate 10, B-1200 Brussels, Belgium; 2 Pôle de Pneumologie, O.R.L. et Dermatologie (LuNS, Lung-Nose-Skin), Institut de Recherche Expérimentale et Clinique, Université Catholique de Louvain (UCLouvain), B-1200 Brussels, Belgium; marylene.lecocq@uclouvain.be (M.L.); bruno.detry@uclouvain.be (B.D.); francois.carlier@chuuclnamur.uclouvain.be (F.C.); charles.pilette@uclouvain.be (C.P.); 3 IREC Imaging Platform (2IP, RRID:SCR_023378), Institut de Recherche Expérimentale et Clinique, Université Catholique de Louvain (UCLouvain), B-1200 Brussels, Belgium; 4 Department of Pathology, Cliniques Universitaires Saint Luc, Université Catholique de Louvain (UCLouvain), B-1200 Brussels, Belgium; 5 Department of Pulmonology, CHU-UCL Namur, B-5530 Yvoir, Belgium; 6 Department of Pulmonology, Cliniques Universitaires Saint Luc, Université Catholique de Louvain (UCLouvain), B-1200 Brussels, Belgium; 7 Department of Cardiovascular and Thoracic Surgery, Cliniques Universitaires Saint Luc, Université Catholique de Louvain (UCLouvain), B-1200 Brussels, Belgium

**Keywords:** airway epithelium, mucosal immunity, immunoglobulin A, hypoxia

## Abstract

Secretion of immunoglobulin A (IgA), actively transported across the airway epithelium (AE) by the polymeric immunoglobulin receptor (pIgR), represents a key defense of the lung against pathogens. Dysregulation of the IgA/pIgR system has been reported in respiratory diseases, but the underlying mechanism, and particularly the effects of hypoxia, remains poorly characterized. The effects of hypoxia were investigated using the model of human AE reconstituted in vitro upon culture in the air/liquid interface (ALI). AE was exposed to 1% O_2_, upon resting or inflammatory conditions. Mechanistic studies included pharmacological stabilization of HIF-1α and inhibition of TGF-β signaling. Additionally, HIF-1α and TGF-β pathways were analyzed by multiplex fluorescence immunostaining of lung tissue from patients with acute respiratory distress syndrome (ARDS) and controls. In ALI AE cultures from 22 subjects, hypoxia significantly reduced pIgR expression (mRNA and proteic), regardless of inflammation. Hypoxia also altered AE structure and cell differentiation, leading to a loss of ciliated and goblet cells. Both hypoxia and pharmacological stabilization of HIF-1α recapitulated these effects and induced increased TGF-β2 expression. In lung tissue from ARDS patients, the AE exhibited reduced pIgR expression, associated with increased HIF-1α stabilization and TGF-β2 expression. Hypoxia induced pIgR downregulation and impaired cell differentiation, through HIF-1α stabilization and subsequent autocrine TGF-β2 signaling, providing a novel pathway underlying altered lung IgA immunity in disease.

## 1. Introduction

The airway epithelium (AE) fulfills multiple functions to maintain pulmonary homeostasis, acting as a first-line barrier against inhaled particles while participating in local innate and adaptative immune mechanisms including control of inflammatory mechanisms [1]. Airway epithelial cells transport immunoglobulin A (IgA), the predominant mucosal immunoglobulin in the airway lumen. IgA, produced by submucosal B-cells in polymeric form, is actively transported from the basolateral to the apical pole of epithelial cells by the polymeric immunoglobulin receptor (pIgR). Cleavage of the pIgR at the apical surface releases its extracellular part, called secretory component (SC), either free or bound to IgA, thereby generating secretory IgA (S-IgA) [2]. In the lumen, S-IgA and SC exert anti-bacterial, anti-viral, pro-inflammatory and immunomodulating functions.

Dysregulation of the mucosal IgA/pIgR system has been reported in several chronic respiratory diseases, such as chronic obstructive pulmonary disease (COPD) [3,4], asthma [5], lung cancer [6], chronic rhinosinusitis [7], idiopathic lung fibrosis (IPF) [8], and, recently, acute respiratory distress syndrome (ARDS) [9]. In the latter, decreased pIgR expression was associated with extensive epithelium injury, including basal membrane denudation and impaired mucociliary differentiation [9].

Although multiple inducers of *PIGR* expression have been identified, including cytokines and microbial products [10,11], the mechanisms underlying pIgR downregulation remain poorly characterized. Recent in vitro studies demonstrated that *PIGR* expression is downregulated by TGF- β, whereas the precise mechanisms remain elusive. Inflammation and hypoxia constitute two hallmarks of several pathological processes in the lung. Hypoxia has been associated with altered epithelium differentiation [12,13], induction of epithelial-to-mesenchymal transition (EMT) [14], and increased barrier permeability [15]. However, the impact of tissue hypoxia on the regulation of the IgA/pIgR system remains unexplored.

The present study aimed to explore the effects of hypoxia, inflammation, and their associated molecular pathways on broncho-epithelial functionality and pIgR expression in vitro in a model of reconstituted human bronchial epithelium derived from controls and cultured in the air–liquid interface (ALI). Additionally, pathways associated with pIgR downregulation were analyzed in situ by multiplex fluorescence immunostaining of lung tissue from patients with acute respiratory distress syndrome (ARDS) and controls.

## 2. Results

### 2.1. Altered pIgR Expression and Epithelium Differentiation in the Model of ALI-Reconstituted AE Exposed to Hypoxia

To delineate the respective roles of hypoxia and inflammation in the observed alterations of the pIgR/SC system and AE differentiation in ARDS (9), human bronchial epithelia derived from control patients cultured in the air–liquid interface (ALI) were submitted either to hypoxia and/or to inflammatory cytokines. Epithelia grown in the hypoxic condition showed a sustained decrease in pIgR protein expression observed after 7 days of hypoxia (Figure 1A,B), while a decrease in apical SC release (Figure 1C) and pIgR mRNA expression (Figure 1D) were already identified after 2 days—and sustained at 7 and 12 days—of culture in hypoxic conditions. Altogether, this indicates a transcriptional downregulation of *PIGR* upon hypoxia.

In addition, a striking dedifferentiation of the AE was observed upon hypoxia (HE staining, Figure 2A), which translated into a sustained decrease in trans-epithelial resistance (TER) (Figure 2B). Hypoxic airway epithelia also showed a decreased expression of E-cadherin and both β-Tubulin and MUC5AC protein expression (Figure 2C–F). A decreased mucociliary lineage differentiation was confirmed by decreased transcription of *FOX J1* (Figure 2G) and SAM Pointed Domain Containing ETS Transcription Factor (*SPDEF*, goblet cells, Figure 2H) in the hypoxic condition, while *P63* (basal cells) and *FOX M1* (club cells) mRNA abundance was not significantly altered (Figure 2I,J).

In contrast, inflammation induced by the pro-inflammatory cytokine mix failed to affect the expression of pIgR/SC (Figure S1A–D, online data Supplementary File) or the differentiation of the AE, even though decreased TER was noticed (Figure S2A–J).

### 2.2. Hypoxia-Induced Alterations in pIgR Expression and Epithelium Differentiation Are Recapitulated by HIF-1α Stabilization

Hypoxia induced the stabilization of HIF-1 α (Figure 3A), which translated into a sustained increase in the expression of glucose transporter 1 (*GLUT1A*, Figure 3C) and Lactate Dehydrogenase A (*LDH-A*, Figure 3D) mRNA, two common HIF target genes. The addition of Dimethyloxalylglycine (DMOG), a chemical HIF-1α stabilizer (Figure 3A) in the basolateral medium of epithelia exposed to 21% of O_2_, recapitulated the findings observed upon hypoxia, namely an increased transcription of HIF-1α target genes (Figure 3B,C) and a decrease in the expression of the pIgR protein (Figure 3D,E) and of the apical release of SC (Figure 3F), confirming that the hypoxic downregulation of pIgR was mediated by HIF-1α. In addition, HIF-1α stabilization induced a decrease in pIgR mRNA expression (Figure 3G), confirming that the effects of hypoxia on pIgR expression involved a transcriptional downregulation.

Furthermore, prolonged (12 days) DMOG exposure mimicked the alterations of AE differentiation observed in hypoxia (Figure 4A–C), with a loss of ciliated (Figure 4D) and goblet cells (Figure 4E), without significant changes in club and basal cell populations (Figure 4F,G).

### 2.3. Hypoxia-Induced AE Damage May Involve TGF-β Signaling

Based on previous studies linking TGF-β to decreased pIgR expression [16] and to hypoxia [17] in AE cells, we sought to determine whether TGF-β was involved in the hypoxic downregulation of pIgR and of epithelial differentiation. Long-lasting exposure of control epithelia to hypoxia or pharmacological stabilization of HIF-1α was associated with increases in total (Figure 5A) and active (Figure 5B) TGF-β2 (measured by ELISA in BL medium), as well as in *TGF-β2* mRNA (Figure 5C), while the effects on TGF-β1 were much less significant (Figure 5D–F). In addition, pharmacological inhibition of TGF-β with 1D11, a mAb neutralizing the three TGF-β isoforms, achieved a partial recovery of pIgR expression (Figure 5G–H) and of SC release (Figure 5I) in hypoxic AE, despite significant heterogeneity.

### 2.4. Increased HIF-1α Stabilization and TGF-β2 Expression in the Airways of Patients with ARDS

Based on our previous work showing epithelium damage and decreased pIgR expression in the airways of patients with ARDS [9], we hypothesized that regional hypoxia and subsequent increased TGF-β2 expression could be involved in pIgR downregulation (and in other epithelial alterations) observed in situ. Therefore, we performed a quantitative evaluation of fluorescence immunostaining of whole tissue sections from ARDS patients (n = 12) and controls (n = 11) (data in Table S2, online data Supplementary File), with costainings for pIgR, p63 (basal cells), Carbonic Anhydrase 9 (CA9, surrogate for hypoxia and HIF-1α stabilization in tissue, Figure 6A) and TGF-β2 (Figure 6E). After confirming pIgR protein downregulation (Figure 6B), multiplex immunofluorescence quantitative analysis also showed a trend towards increased expression of CA9 (Figure 6C, 1.47% [0.78–11.48] vs. 0.79% [0.15–2.39], *p* = 0.0597). More specifically, the expression of CA9 was significantly higher in pIgR+ epithelial areas (Figure 6D, 3.23% of pIgR+ area [1.27–9.31] vs. 0.83% [0.13–1.37], *p* = 0.0036). In addition, there was an increased expression of TGF-β2 (Figure 6F, 1.84% [0.25–8.46] vs. 0.17% [0.10–1.50], *p* = 0.023) and an increased co-expression of TGF-β2 and pIgR (Figure 6G, 4.69% of pIgR+ area [0.732–15.81] vs. 0.30 [0.060–1.58], *p* = 0.016) in the AE of ARDS patients.

## 3. Discussion

This study demonstrates that hypoxia—but not inflammatory cytokines—induces a transcriptional downregulation of pIgR and altered epithelium differentiation in primary cultures of human ALI-AE. It also suggests that the underlying mechanisms involve stabilization of HIF-1α and increased TGF-β2 signaling. In addition, it reveals that lung tissue from patients with ARDS consistently displays HIF-1α stabilization and elevated TGF-β2 expression, suggesting that hypoxia may contribute to alterations of pIgR-related IgA immunity and of the epithelium barrier in these patients.

Multifaceted alterations of the AE—such as epithelial dedifferentiation, increased barrier permeability, and dysregulation of the IgA/pIgR system—are hallmarks of various pathological processes in the lung [8,9,16]. Notably, AE cells are exposed to hypoxia during such conditions [18,19]. Therefore, the mechanisms of AE alterations could involve local inflammation and/or hypoxia, both of which have demonstrated effects on epithelium permeability [20,21,22] or differentiation [1,12,13,23] in vitro. Using the validated model of ALI-AE [16], we demonstrate that sustained hypoxia stabilizes HIF-1α and leads to transcriptional downregulation of the *PIGR*, which translates into reduced expression of the pIgR protein and reduced secretion of SC in the apical supernatants. This finding reveals a novel mechanism by which hypoxia may compromise innate immune defenses in the lung by disrupting the IgA/pIgR system. The impact of hypoxia on the AE was recently studied by Mikami et al. in the ALI-AE model, who reported decreased *PIGR* mRNA levels upon hypoxia, a finding consistent with our study [23]. In addition to its effects on the IgA/pIgR axis, hypoxia is known to impair other epithelial defense mechanisms, including increased permeability [22], downregulation of the secretory leukocyte protease inhibitor (SLPI) via a TGF-β-dependent pathway [24], inhibition of Pseudomonas aeruginosa internalization [25] and direct effects on pathogens [26,27,28], all of which may contribute to the complex interplay between host defense and microbial pathogenicity [29].

In our study, AE grown under the hypoxic condition shows morphologic alterations, decreased trans-epithelial resistance and reduced mucociliary lineage differentiation, involving a decrease in both ciliated and goblet cells. Although a decreased number of ciliated cells was previously observed in a similar model of hypoxia [13], another study found that sustained hypoxia and HIF-1α stabilization induced goblet cell hyperplasia and increased expression of MUC5AC [12]. Conversely, a recent study using a model of primary ALI-AE exposed to hypoxia for a shorter period (5 days) reported no significant changes in AE morphology or cell-type distribution under hypoxia [23]. These apparent discrepancies may be attributed to differences in experimental protocols, including the timing of hypoxia exposure (applied after complete epithelium differentiation in our study), the duration of hypoxia or the pharmacological agents used for HIF-1α stabilization. This also suggests that the effects of hypoxia on AE structure and function might be heterogeneous in vivo.

Further experiments conducted in ALI AE revealed that chemical stabilization of HIF-1α using DMOG replicated the findings observed under hypoxia, including decreased pIgR expression and altered AE differentiation. In addition, we observed an increase in TGF-β2 expression in hypoxic AE as well as after HIF-1α stabilization, and pharmacological inhibition of TGF-β led to a partial recovery of pIgR expression. However, it should be noted that the pharmacological inhibition of TGF-β was not specific to the β2 isoform, that it was performed on a limited number of samples (n = 5), and that pIgR increase after TGF-β signaling did not reach statistical significance due to significant heterogeneity. Therefore, based on our data, the proposed mechanistic link between hypoxic AE alterations and TGF-β signaling should be considered exploratory. However, this hypothesis is further supported by previous studies linking TGF-β to hypoxia [24] and to decreased pIgR expression [16] or alveolar epithelium dedifferentiation [30]. Future research should delineate the precise molecular pathways.

In previous work [9], we observed similar alterations in AE differentiation and decreased pIgR expression in the airways of patients with acute respiratory distress syndrome (ARDS), leading to compromised defense functions and increased permeability. ARDS is often associated with infection (systemic and/or pulmonary), uncontrolled inflammation, airway obstruction, alveolar edema, and atelectasis. These factors collectively contribute to the profound imbalance between oxygen supply and demand, making tissue hypoxia central in the pathogenesis of ARDS [31]. However, delineating the respective contributions of hypoxia and inflammation to epithelium alterations and lung immune responses is particularly challenging in the context of ARDS, as both processes are closely intertwined. In particular, the role of HIFs and the potential effects of HIF modulation are complex and seem primarily dependent on the clinical context [32]. While HIF activation/modulation has never been explored in clinical studies involving ARDS patients, experimental models of acute lung injury provide conflicting results regarding the adaptative or maladaptive role of HIF activation during lung injury [33]. It is therefore likely that the roles of HIF-1α are disparate, depending on factors such as cell type, nature of the inflammatory response, and timing of injury [33]. Based on our in vitro observations, we hypothesize that hypoxia and HIF-1α stabilization, followed by autocrine TGF-β signaling, contribute to the observed alterations in AE of patients with ARDS. This hypothesis is supported by the observed increase in Carbonic Anhydrase 9 (CA9, a marker of HIF-1α gene activation and a surrogate marker for tissue hypoxia [34]) and TGF-β2 expression in pIgR+ regions of the airways in ARDS patients. This suggests that HIF-1α is specifically stabilized, and TGF-β2 is induced, in AE cells expressing pIgR exposed to hypoxia.

Our study has several limitations. First, in the experimental model of reconstituted AE, the partial pressure of oxygen was deliberately set at 1% in the hypoxic group and 21% in the normoxic group. While this is in line with most experimental models [12,13,24,35], we did not measure oxygen tension in vivo in control or diseased AE and we cannot exclude that these conditions applied in vitro may significantly differ from the actual pO_2_ of the AE in vivo, which is largely speculative [36]. Second, DMOG, the prolyl hydroxylase inhibitor used to achieve stabilization of HIF-1α, is involved in other metabolic pathways, and we cannot exclude that some of the observed alterations in AE structure or function could have been caused by off-target, nonspecific effects. Third, it is not clear whether inhibiting HIF-1α activity could constitute a promising therapeutic target, given the multiple pathways in which it is involved. Therefore, a more comprehensive view of the signaling pathways (downstream of HIF-1α) engaged in the AE during hypoxia should be obtained. Fourth, although we demonstrate that a mix of inflammatory mediators did not affect pIgR expression or AE differentiation, we cannot exclude the possibility that other inflammatory pathways may influence pIgR expression and AE structure in vivo. Lastly, hypoxia markers in the airways of ARDS patients were compared in vivo with those in controls but several important differences between ARDS and control groups need to be acknowledged. The mode of tissue acquisition differed between ARDS and controls (open lung biopsy for the former and lobectomy for the latter), potentially influencing the expression of epithelial makers. Moreover, controls were younger than ARDS, and lobectomy was performed in controls for a solitary lung tumor, which may also have affected the expression of some epithelial markers, such as pIgR, whose expression seems to differ between cancer types [6,37]. It should be noted that most controls (10/11) underwent lobectomy for a solitary colorectal cancer metastasis, whereas the remaining control had a small solitary primary lung cancer. In all cases, lung tissue was sampled at the greatest possible distance from the lesion to minimize the potential influence of the tumor on epithelial marker expression. Nevertheless, given these important potential confounding factors, these in vivo findings suggesting increased expression of hypoxia markers and activation of TGF-β pathways in patients with ARDS should be interpreted with caution and considered exploratory.

## 4. Material and Methods

### 4.1. In Vitro Reconstitution of Primary Human Bronchial Epithelium in ALI Culture

More details regarding the reagents used can be found in the online data Supplementary File (Tables S1–S3).

A large piece of lobar or segmental bronchus (3rd or 4th generation) was selected from lobectomies or lung explants of control patients, without evidence of diffuse respiratory disease (*n* = 22), located as far as possible from the tumor site (in the case of lobectomies) or randomly selected in the case of unused explants from donors (rejected for a pulmonary-unrelated reason). Tissue samples were submitted to pronase digestion to derive primary human broncho-epithelial cells (HBECs) for each sample, which were processed to reconstitute functional bronchial epithelium in ALI culture (details in the online data Supplementary File). The culture was then carried out in ALI until full differentiation (4 weeks in ALI). Culture medium was changed every 48 h.

Fully differentiated airway epithelia were submitted or not to hypoxia and/or to inflammatory cytokine treatment. Hypoxia (1%O_2_, 5%CO_2_, 94% nitrogen) was induced by setting ALI cultures (*n* = 22) in a hypoxic sealed chamber (InVivo 400 Worksation, Ruskinn LTD, Bridgend, UK) for up to 12 days. Inflammation was induced by a pro-inflammatory cytokine mix (*n* = 15) consisting of IL-1β, IL-6, and TNF-α, each at 2.5 ng/ml (Miltenyi Biotec, Bergisch Gladbach, Germany) in the basolateral compartment, based on a preliminary titration experiment (10, 5, and 2,5 ng/ml). For HIF-1α stabilization under normal oxygen tension, Dimethyloxaloylglycine (DMOG) was used (SC, CAS 89464-63-1). DMOG was dissolved in Phosphate-Buffered Saline (PBS), diluted for a final concentration of 1 mM after a titration experiment (0.1 mM and 1 mM, not shown), and added in the basolateral medium of selected inserts (*n* = 9). For TGF-β blocking experiments under hypoxic conditions, a TGF-β blocking antibody (Monoclonal Mouse IgG1 Clone #1D11, MAB1835-500, R&D systems, Minneapolis, MN, USA) was used diluted in PBS at a final concentration of 1.25 μg/ml (after a preliminary titration experiment at 0.25 and 1.25 μg/ml) and added in the basolateral medium of selected inserts (*n* = 5).

After 2 days, 7 days and 12 days of ALI culture in hypoxic/normoxic, stimulated/non-stimulated conditions, basolateral media were collected, and the apical pole of HBECs was washed with 300 µl sterile PBS before centrifugation for 5 min at 10,000× *g*. Transwell inserts were fixed by direct immersion in 4% buffered formaldehyde, before incubation in PBS (pH 7.4) and embedding in paraffin blocks. Secretory component (SC) and Transforming Growth Factor-β (TGF-β) were measured in apical washes by enzyme-linked immunosorbent assays (ELISAs, details in the online data Supplementary File). ALI-HBECs were also processed for mRNA abundance or Western blot analyses (details in the online data Supplementary File). Trans-epithelial electric resistance (TER) was assessed at the same timepoints for each sample using the EMD Millipore™ Millicell-ERS Volt-Ohmmeter (Fisher Scientific, Hampton, NH, USA) after transiently filling the apical pole with sterile PBS and correcting for the resistance of the transwell membrane.

### 4.2. Multiplex Immunostaining of Lung Tissue from ARDS Patients

Lung tissue was obtained from open lung biopsies by surgical wedge resection from patients with ARDS [38], performed for non-resolving ARDS or for ARDS with unknown etiology. The control group consisted of lung tissue from patients who had undergone lobectomy for a solitary lung tumor, with sections taken at maximal distance from the lesion. Patient characteristics can be found in Supplementary Table S2 (online data Supplementary File). Details regarding multiplex immunofluorescence (mIF) staining, image acquisition and quantitative evaluation can be found in the online data Supplementary File.

### 4.3. Statistical Analysis

Statistical analyses were performed using SPSS 21 (IBM SPSS Statistics for Windows, Version 21.0. Armonk, NY, USA) and figures were created using Graphpad Prism 10 (GraphPad Software, version 10.6. LaJolla, CA, USA). Values were expressed as median (interquartile range [IQR]) unless otherwise stated. Categorical and continuous variables were analyzed using the chi-square test and Mann–Whitney or Wilcoxon (paired samples) test, respectively. All tests were two-sided, with a significance level set at 0.05.

## 5. Conclusions

Overall, this study indicates that hypoxia induces pIgR downregulation and airway epithelium dedifferentiation in vitro and may play an important role in AE damage and in alterations of lung immune responses in vivo. Moreover, our work suggests that these hypoxic AE alterations are associated with HIF-1α stabilization and increased TGF-β2 signaling. Therefore, HIF-1α and downstream pathways may constitute promising novel therapeutic targets to restore lung epithelium defense in such pathological settings.

## Figures and Tables

**Figure 1:**
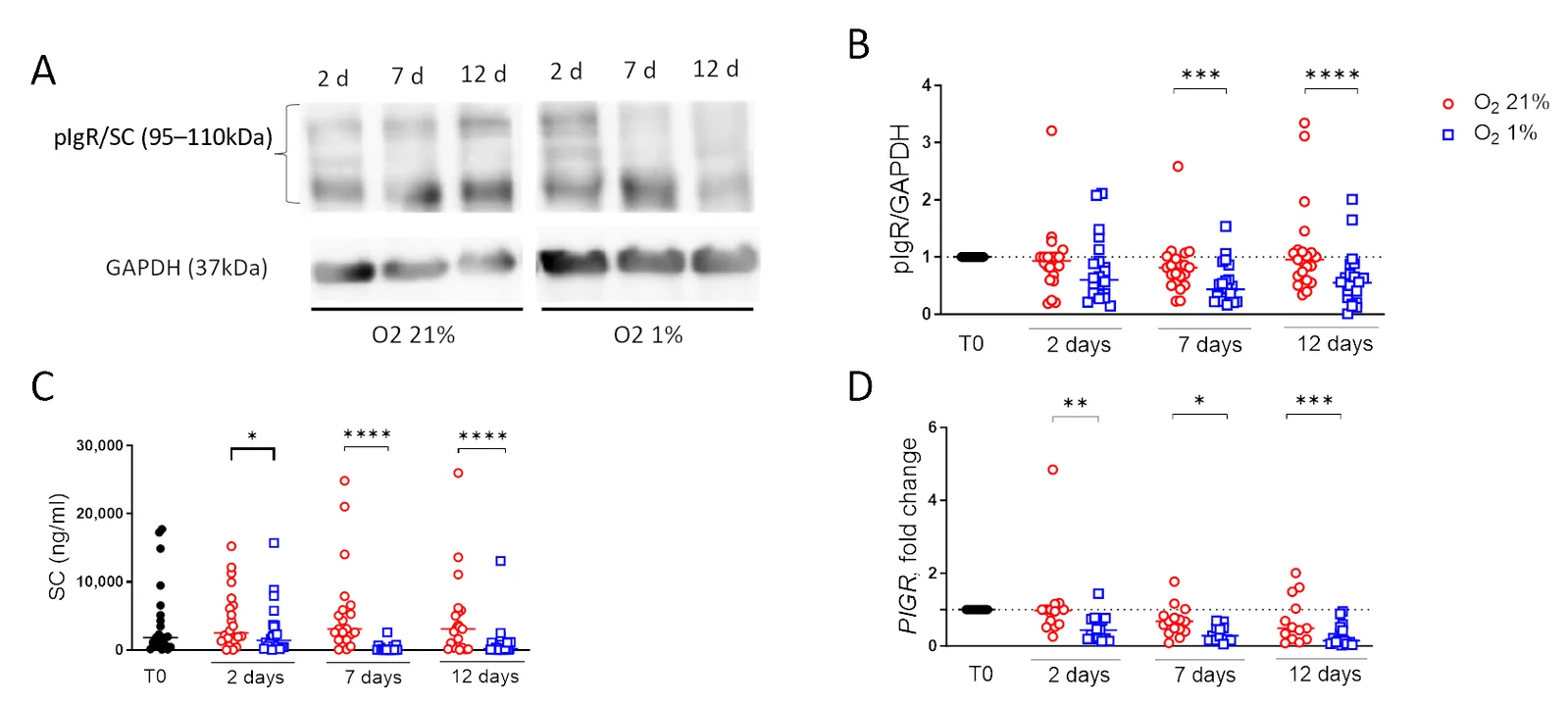
**Hypoxic downregulation of pIgR in control AE.** (**A**) Representative blot for pIgR and GAPDH in AE from one control cultured under 21% O_2_ (normoxia, left) or 1% O_2_ (hypoxia, right) for 2, 7 or 12 days. (**B**) Quantification of pIgR expression in ALI AE cultured upon normoxia (red dots) or hypoxia (blue dots) at baseline and after 2, 7 and 12 days, normalized for GAPDH (*n* = 22 control AE). (**C**) SC release in apical washes measured by ELISA (*n* = 22). (**D**) Quantification of pIgR mRNA expression, measured by RT-qPCR and normalized for housekeeping (HK) genes (*n* = 14). Between-group difference was evaluated using the Wilcoxon test; * indicates *p* < 0.05, ** *p* < 0.005, *** *p* < 0.0005, **** *p* < 0.00005.

**Figure 2:**
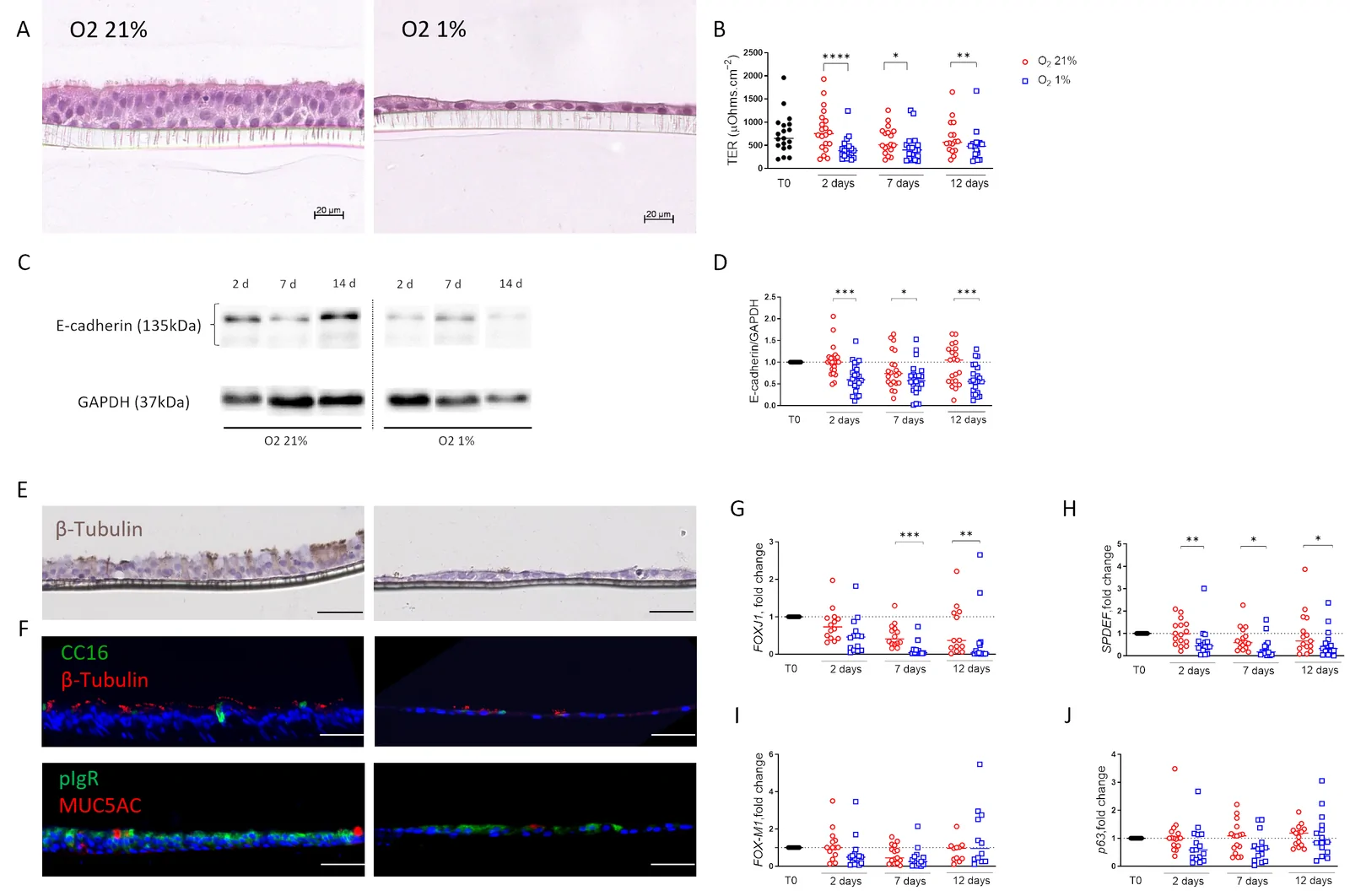
**AE structure alterations induced by hypoxia**. (**A**) Representative Hematoxylin/Eosin-stained ALI-AE from a control, cultured for 12 days under normoxia (left) or hypoxia (right). (**B**) Decreased trans-epithelial electric resistance (TER) in AE from controls (*n* = 20) in hypoxia or normoxia. (**C**,**D**) Representative blot for E-cadherin and GAPDH (c) and quantification of E-cadherin expression in ALI-AE cultured under normoxia or hypoxia at baseline and after 2, 7 and 12 days (D, *n* = 22). (**E**,**F**) Representative immunohistochemistry (**E**) and immunofluorescence (**F**) of AE from one control, cultured for 12 days under normoxia (**left**) or hypoxia (**right**), suggesting decreased β-Tubulin (**E**,**F**), pIgR and Muc5AC (**F**) expression upon hypoxia. Scale bars, 50 µm. (**G**–**J**) Quantification of the mRNA expression of FOXJ1 (epithelial ciliated cells, **G**), SPDEF (goblet cells, **H**), FOXM1 (club cells, **I**) and p63 (basal cells, **J**) measured by RT-qPCR (*n* = 14) and normalized for housekeeping (HK) genes. Between-group difference was evaluated using the Wilcoxon test; * indicates *p* < 0.05, ** *p* < 0.005, *** *p* < 0.0005, **** *p* < 0.0001.

**Figure 3:**
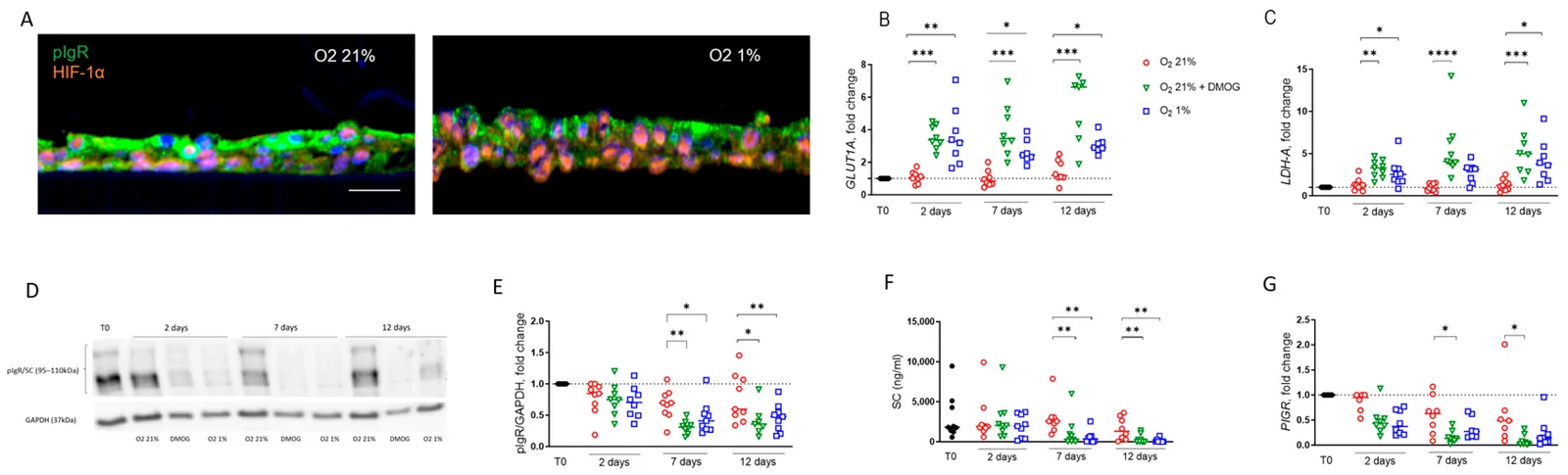
**Hypoxia-induced loss of pIgR expression is mediated by HIF-1α stabilization.** (**A**) Representative immunofluorescence staining of a control airway epithelium, showing increased HIF-1α expression in hypoxic condition. (**B**,**C**) Increase in the mRNA expression of GLUT1A (**B**) and LDH-A (**C**) in airway epithelium cultured under 1% O_2_ or under 21% O_2_ with DMOG compared to control conditions (*n* = 9). (**D**,**E**) Representative blot for pIgR and GAPDH (**E**) and quantification (**F**) of pIgR expression in control airway epithelium cultured under 21% O_2_, 21% O_2_ with DMOG or 1% O_2_ for up to 12 days, showing a decrease in pIgR upon exposure to DMOG or hypoxia after 7 and 12 days (*n* = 9). (**F**) Decrease in SC release in apical washes measured by ELISA upon hypoxia and exposure to DMOG. (**G**) Decrease in pIgR mRNA expression upon exposure to DMOG. Between-group difference was evaluated using the Wilcoxon test; * indicates *p* < 0.05, ** *p* < 0.005, *** *p* < 0.0005, **** *p* < 0.0001.

**Figure 4:**
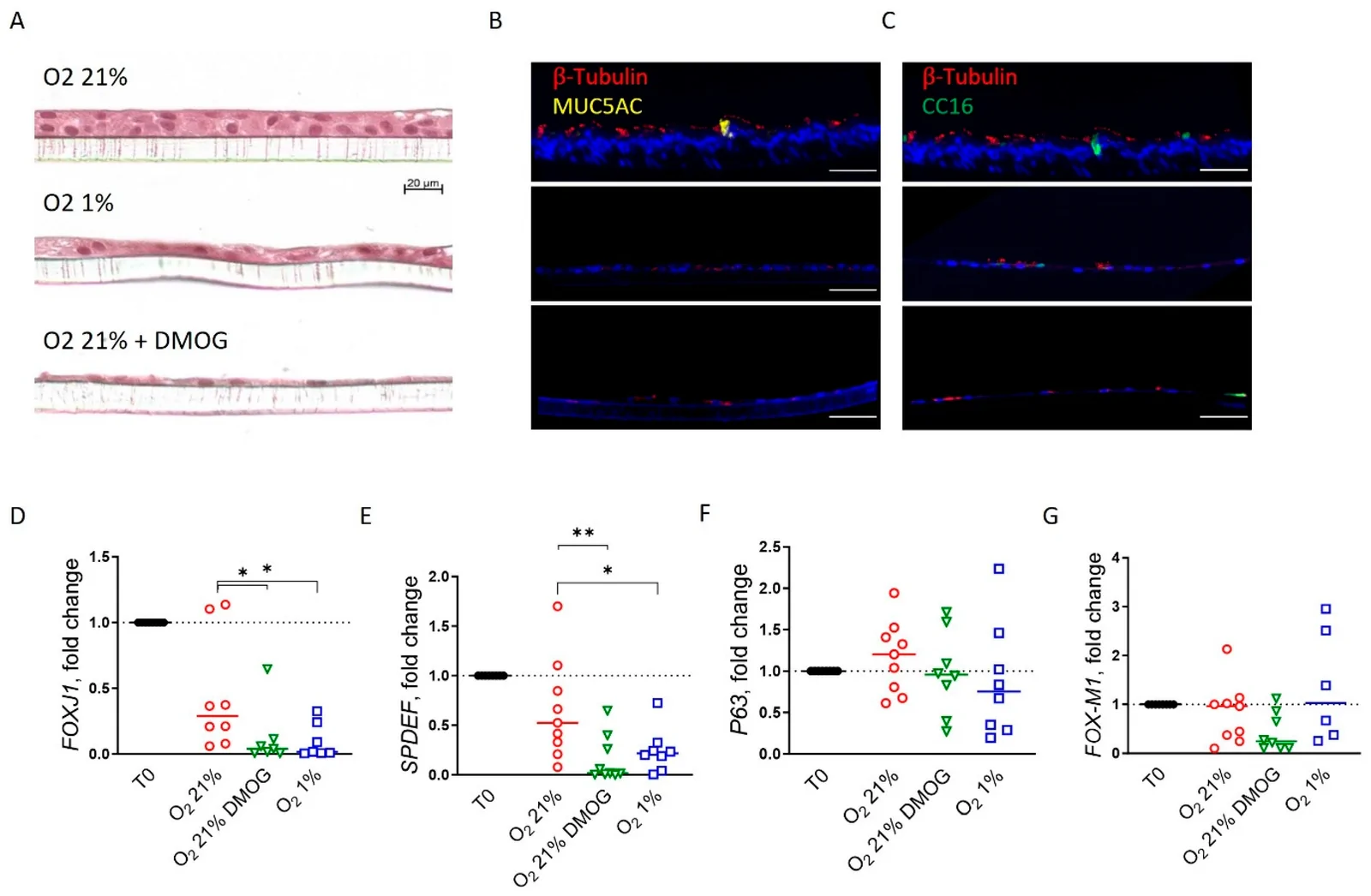
**HIF-1α stabilization effects on AE structure.** (**A**–**C**) Representative Hematoxylin/Eosin-stained (**A**) or immunofluorescence with costainings for β-Tubulin and MUCAC (**B**) or with β-Tubulin and CC16 (**C**) in the AE from one control, grown for 12 days under 21% O_2_ (upper panel), 1% O_2_ (middle panel) or 21% O_2_ with Dimethyloxalylglycine (DMOG; lower panel). Scale bars, 50 µm. (**D**–**G**) There was a decrease in the mRNA expression of *FOXJ1* (**D**) and *SPDEF* (**E**), whereas *P63* (**F**) and *FOXM1* (**G**) remained unchanged in AE grown under 1% O_2_ or under 21% O_2_ with DMOG compared to control conditions (*n* = 8). Data are median ± IQR. Between-group difference was evaluated using the Wilcoxon test; * indicates *p* < 0.05, ** *p* < 0.005.

**Figure 5:**
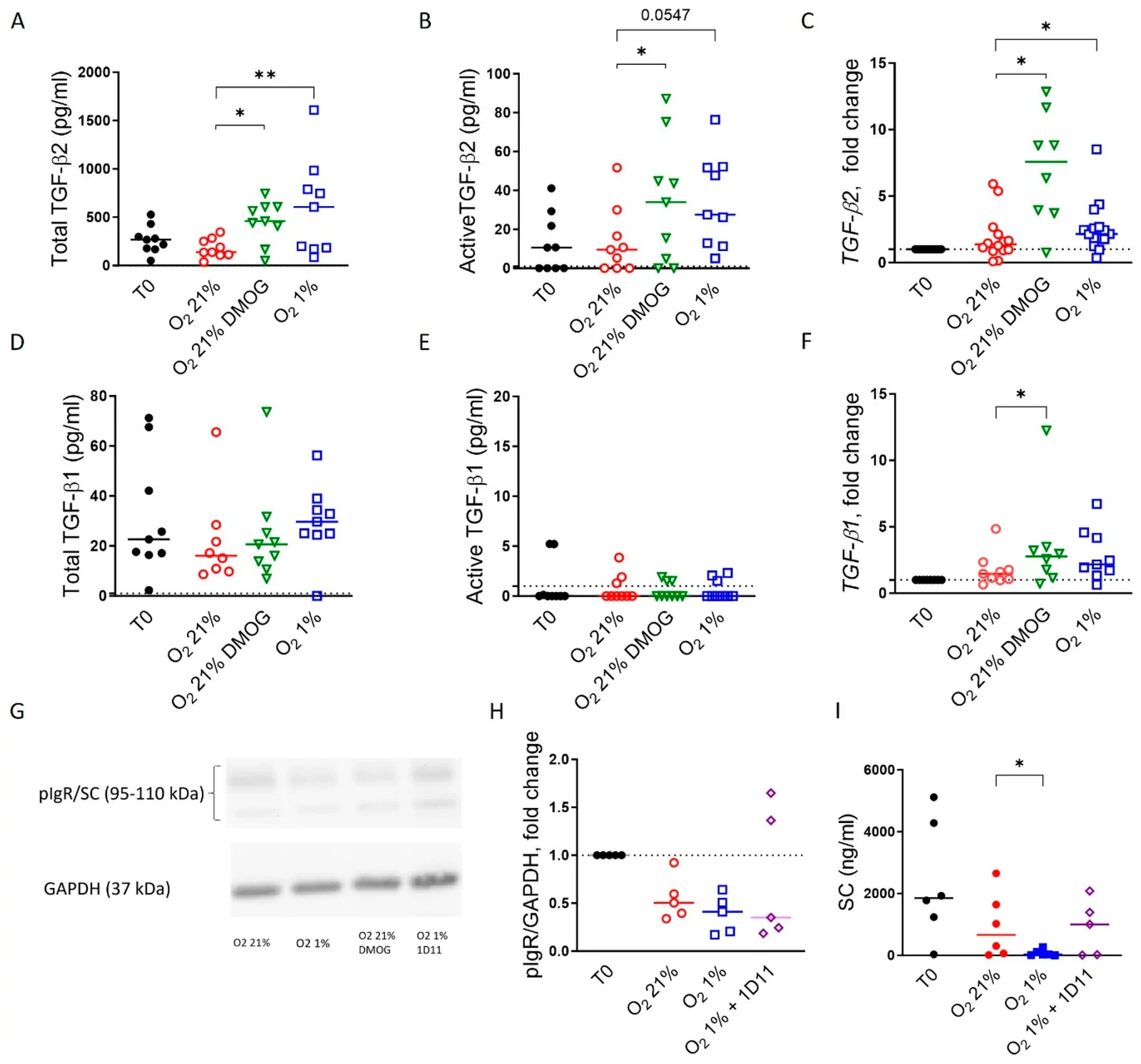
**TGF-β2 is involved in the hypoxia-induced AE damage and downregulated pIgR expression in vitro.** (**A**–**C**) Long-lasting (12 days) hypoxia and HIF-1α stabilization with Dimethyloxalylglycine (DMOG) are associated with significant increases in total (**A**) and active (**B**) TGF-β2 (measured by ELISA in BL medium, *n* = 9), as well as an increase in TGF-β2 mRNA (**C**). (**D**–**F**) In contrast, only a slight increase in TGF-β1 mRNA was associated with HIF-1α stabilization with no significant change in total and active TGF-β1. G-I. Representative blot for pIgR and GAPDH (**G**) and quantification of pIgR expression (**H**) and of SC release in the apical washes (**I**) of AE cultured for 12 days under 21% O_2_, 1%O_2_ or 1% O_2_ with 1D11, a neutralizing antibody for all TGF-β isoforms, suggesting partial recovery of pIgR expression and of SC release in hypoxic AE treated by 1D11 (*n* = 5). Data are median ± IQR. Between-group difference was evaluated using the Wilcoxon test; * indicates *p* < 0.05, ** *p* < 0.005.

**Figure 6:**
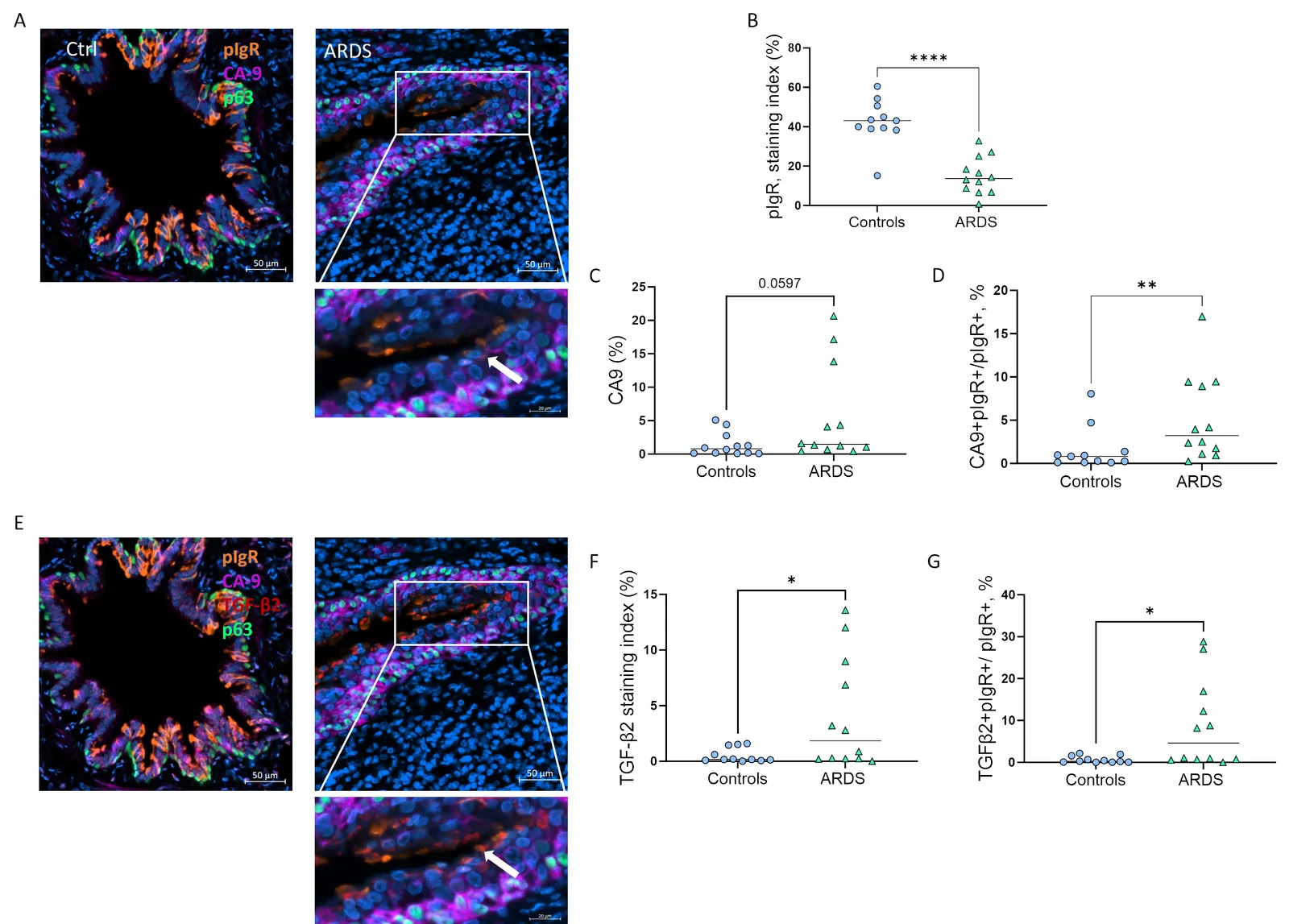
**Hypoxia markers and TGF-β expression in the AE of patients with ARDS compared to controls.** (**A**) Representative immunofluorescence staining of lung tissue sections from a control (**left**) and an ARDS patient (**right**) stained with Carbonic Anhydrase-9 (CA9) and pIgR, costained with p63 and Hoechst (lower panel with high magnification; white arrow showing a cell co-expressing CA9 and pIgR). Scale bars, 50 µm (20 µm for the high magnification panel). (**B**) Quantification of the area expressing pIgR (in proportion of the total AE area), showing a significant decrease in the expression of pIgR expression within the AE of ARDS patients (*n* = 12) compared to controls (*n* = 11). (**C**) Quantification of the area expressing CA9, showing a non-significant trend towards increased expression of CA9, reflecting AE hypoxia in patients with ARDS. (**D**) Quantification of the area co-expressing pIgR and CA9 (expressed in proportion of the total pIgR area) showing an increased CA9 expression within airway epithelial cells expressing pIgR in the same ARDS patients and controls. (**E**) Representative immunofluorescence staining of the same sections from a control (**left**) and an ARDS patient (**right**) stained with TGF-β2, costained with CA9, pIgR, p63 and Hoechst (lower panel with high magnification; white arrow showing a cell co-expressing TGF-β2 and pIgR). Scale bars, 50 µm (20 µm for the high magnification panel). (**F**) Quantification of the area expressing TGF-β2, showing an increased expression of TGF-β2 in the airways of patients with ARDS. (**G**) Increase in TGF-β2 expression in AE cells expressing pIgR in ARDS patients. Between-group difference was evaluated using the non-parametric Mann–Whitney U test; * indicates *p* < 0.05, ** indicates *p* < 0.005, **** indicates *p* < 0.0001.

## Data Availability

The datasets used and/or analyzed during the current study are available from the corresponding author on reasonable request.

## Supplementary Materials

The following supporting information can be downloaded at: https://www.mdpi.com/article/10.3390/ijms27219525/s1.
