# Supplementary material for: Hypoxia Alters Polymeric Immunoglobulin Receptor Expression and Cell Differentiation in the Airway Epithelium
Source: Int J Mol Sci. 2026 Oct 24;27(21):9525. doi: 10.3390/ijms27219525 (PMC13763201; doi:10.3390/ijms27219525)
Supplement: Supplementary file 1 [file ijms-27-09525-s001.zip › ijms-4553912-supplementary.pdf]

Hypoxia Alters Receptor-Mediated IgA Transport and Cell Differentiation in the Airway Epithelium

Authors:

Ludovic Gerard [1,2,*] , Marylene Lecocq [2], Bruno Detry [2], Caroline Bouzin [3], Delphine Hoton [4], François Carlier [2,5], Thomas Plante-Bordeneuve [2,5], Sophie Gohy [2,6], Valérie Lacroix [7] and Charles Pilette [2,6]

Online data supplement

METHODS

In vitro reconstitution of primary human bronchial epithelium in ALI culture

Tissue samples were submitted to pronase digestion overnight at 4°C, to derive primary human broncho-epithelial cells (HBEC) for each sample. HBEC were seeded in 75cm² flasks (2x10^6 alive cells/flask), then cultured in retinoic acid-supplemented Bronchial Epithelial Cell Growth Basal Medium (BEBM, Lonza, Verviers, Belgium) until confluence. Cells were then detached (passage 1), assessed for viability and seeded at a density of 80,000 alive cells/well on 24-well polyester filter-type inserts (0.4-µm pore size; Corning, Corning, NY) coated with 0.2 mg/ml collagen IV (Sigma-Aldrich, Saint-Louis, MO) until a confluent monolayer was obtained. The culture was then carried out in ALI until full differentiation (4 weeks in ALI). Culture medium was changed every 48 hours.

Once in ALI, HBEC were cultured in BEBM : Dulbecco's Modified Eagles Medium (DMEM) (1:1) medium supplemented with penicillin (100U/ml), streptomycin (100µg/ml) (Lonza, Verviers, Belgium), bovine serum albumin (BSA) (1.5 µg/ml), retinoic acid (30ng/ml) (Sigma, Saint-Louis, MO), and BEGM SingleQuotsTM Supplements and Growth Factors (Lonza), including bovine pituitary extract (52µg/ml), insulin (5µg/ml), hydrocortisone (0·5g/ml), transferrin (10µg/ml), epinephrine (0·5µg/ml), epidermal growth factor (0·5ng/ml) and triiodothyronine (3·25ng/ml).

Enzyme Linked immunosorbent Assays for Secretory Component (SC)

96-well plates were coated overnight with anti-human IgA (ACP17 Ab, homemade) or anti-SC (Goat 606, homemade). Blocking was performed with phosphate-buffered saline (PBS) and 1% bovine serum albumin (BSA) prior to the addition of 50µL of sample or standard reagent. SC was detected using a biotinylated anti-SC antibody (homemade). Revelation was performed using 3,3′,5,5′-tetramethylbenzidine (Thermo Fisher Scientific, UK) and stopped with H_2SO_4

1.8 M. Data acquisition was performed on a iMark microplate reader (Biorad, Hercules, CA, USA).

TGF-β1/β2 assays

TGF-β1 and TGF-β2 were measured in the supernatant of Air-liquid interface Human Bronchial Epithelial Cells (ALI AEs) using R&D Human TGF-β1 and Human TGF-β2 duosets (DY240 and DY 302 respectively). Measurements were made directly on the samples for active TGF-β1/2. Conversely, the latent forms of TGF-β1/2 were measured following an acid activation and neutralization protocol in agreement with the manufacturer's instructions. This acidification induced an activation of the latent form of TGF-β1/2 and allowed the measure of the both latent and active forms (total TGF-β1/2).

Reverse transcriptase quantitative polymerase chain reaction (RT-qPCR)

Total RNA was extracted from reconstituted ALI cultured epithelia using TRIzol reagent (Thermo Fisher Scientific). 500ng of RNA was reverse-transcribed with RevertAid H minus Reverse transcriptase kit with 0·3 µg of random hexamer, 20U of RNase inhibitor and 1mM of each dNTP (Thermo Fisher Scientific, Waltham, MA) following the manufacturer's protocol in a thermocycler (Applied Biosystems, Foster City, CA). The expression levels were quantified by real-time quantitative PCR with the CFX96 PCR (Bio-Rad, Hercules, CA). The reaction mix contained 2.5 µl of complementary desoxyribonucleic acid diluted 10-fold, 200nM of each primer (primers properties are detailed in Table S2) and 2x iTaq UniverSybr Green® Supermix (Bio-Rad) in a final volume of 20 µl. The cycling conditions were 95°C for 3 minutes followed by 40 cycles of 95°C for 5 seconds and 60°C for 30 seconds. To control the specificity of the amplification products, a melting curve analysis was performed. The copy number was calculated from the standard curve. Data analysis was performed using Bio-Rad CFX software

(Bio-Rad). Expression levels of target genes were normalized to the geometric mean of the values of 3 housekeeping genes (RPL27, RPS13, RPS18).

Western blot

Tissues were lysed by homogenization in in Laemmeli's buffer and β-mercaptoethanol and stored at – 20°C until further use. All samples were then boiled at 95°C for 5 minutes. Lysates' proteins were separated on a 12% sodium °C dodecyl sulfate-polyacrylamide gel before being transferred on a nitrocellulose membrane. The membranes were incubated during 1 hour with a blocking solution consisting of tris-buffered saline (TBS), 5% bovine serum albumin (BSA) and 0·1% Tween 20 (Sigma-Aldrich) before washing and incubation with the primary antibody (see Table S1 for a list of used antibodies) overnight. Membranes were then washed and incubated with the adequate Horseradish peroxidase (HRP)-linked secondary antibody (Table S3). Immunoreactive bands were revealed using ECL Prime Western Blotting Detection chemiluminescent reagent (GE Healthcare, UK) and visualized using a Chemidoc XRS system (Biorad). Analysis of the obtained images was performed using Quantity One software (Biorad).

Multiplex immunofluorescence (mIF) staining

Lungs were fixed for 24h at 4°C in 4% formaldehyde, embedded in paraffin and sectioned. After deparaffinization, 5 µm tissue sections were processed according to the protocol described by Aboubakar et al (17). After deparaffinization in toluene and methanol, endogenous peroxidases were inhibited for 15 min by adding Bloxall (Vector Laboratories, Peterborough, UK) followed by 30 min treatment with 0·3% hydrogen peroxide. Sections were then submitted to antigen retrieval in 10 mM citrate pH 6.0 buffer containing 0.1% triton (5 min 15 psi) and to blocking of specific antigen-binding sites (Tris buffered saline (TBS) containing 5% normal

goat serum and 0·1% Tween 20). The first primary antibody was incubated in TBS containing 5% normal goat serum and 0·1% Tween 20 and detected by corresponding horseradish peroxidase (HRP)-conjugated polymer secondary antibodies for 40 min at room temperature (RT). HRP was then visualized by tyramide signal amplification (TSA) using AlexaFluor-conjugated tyramides (Thermo Fisher Scientific, Paisley, UK). After a new citrate buffer incubation step (this step also detaches antibodies to the tissue section), the same protocol was applied with other primary antibodies and different AlexaFluor-conjugated tyramides. Details regarding the antibodies used and their concentration can be found in Table S3. After a washing step in PBS, nuclei were finally stained with Hoechst 33342 (Thermo Fisher Scientific) diluted in TBS containing 10% BSA and 0·1% Tween 20, washed in distilled water, and mounted with Dako fluorescence mounting medium (Agilent). Slides were stored at −20 °C until multispectral image acquisition.

Imaging and quantitative evaluation of immunostaining in whole tissue sections

Multiplex immunostained slides were digitalized in fluorescence using a Axioscan.z1 (Zeiss, Germany) slide scanner at x20 magnification. Stainings were quantified on entire tissue sections with software applications ("APP"s) using the image analysis tool Author version 2017.2 (Visiopharm, Hørsholm, Denmark). Bronchi were manually delineated along the basal membrane and were labeled as “Region of Interest (ROI)”. This ROI was then automatically restricted to the epithelium (lumen exclusion) using a thresholding classification method based on all stainings. Manual correction was applied when required to remove blur areas, damaged tissue and artefacts. Due to technical limitations, mainly linked to tightly packed nuclei within epithelia (even worse in ARDS displaying heavily disturbed architecture) and cytoplasmic staining (MUC, beta-tub) far away from the nucleus of the corresponding cell, complicating both cell segmentation and cell classification, we could not quantify stained cells as a proportion

of all tissue cells. Therefore, each staining was detected at high resolution (x20) using a thresholding classification method on each fluorescence channel. Thresholds were adjusted on representative stained vs not stained regions. Results were expressed as a percentage of stained pixels within the region of interest (airways epithelium).

The same strategy was applied for co-localization studies, for example the co-localization of CA-9 or TGF-β2 in airway epithelial cells expressing pIgR. We used a thresholding classification method based on two fluorescence channels. Results were expressed as percentage of CA-9/pIgR or TGF-β2/pIgR co-stained pixels among pIgR-stained pixels [(CA9+pIgR+/pIgR+ area)*100 or (TGF-β2+pIgR+/pIgR+ area)*100]. The same parameters were kept constant for all slides. All analyses were performed by an experienced observer, who was blinded to the clinical categories of the tissue samples.

Table S1. Reagents used for Western Blot.

Antigen	Primary antibody			Secondary antibody
	Dilution	company	Catalog number	HRP
pIgR	1/5000	Home made	Lp877	Anti-rabbit (Cell Signaling 7074S 1/2000)
E-Cadherin	1/1000	Dako	M3612	Anti-mouse
GAPDH	1/5000	Sigma	G9545	Anti-rabbit

Table S2. Baseline characteristics, adjunctive treatments and outcomes for ARDS (n=12) and controls (n=11), for quantitative evaluation of fluorescence immunostaining in whole tissue sections.

Data are presented as count (%), or median (IQR)

Variables	Controls (n=11)	ARDS (n=12)	p
Age, years, median (IQR)	55 (47-68)	70·1 (52·5-78·4)	0·054
Male, n (%)	4 (36)	4 (33·3)	0·59
Diabetes, n (%)	1 (9)	3 (25)	0·43
Active smoking, n (%)	4 (36)	3 (25)	0·31
Chronic obstructive pulmonary disease, n (%)	0	1 (8·3)	0·58
Other chronic respiratory disease, n (%)	0	2 (16·6)	0·32
Active neoplasia, n (%)	11 (100)	4 (33·3)	<0·001
Type of tissue sampling, n (%)			<0·0001
Open-Lung Biopsy	0	12 (100)	
Surgical lobectomy	11 (100)	0	
APACHE II, median (IQR)		16 (11-19)	
SOFA, median (IQR)		5 (3-8)	
Cause of ARDS, n (%)			
COVID-19		0	
Pneumonia		6 (50)	
Sepsis (of extra-pulmonary origin)		1 (8·3)	
Other		5 (41·7)	
Adjunctive treatments before tissue sample, n (%)			
Prone positioning		3 (25)	
Inhaled Nitric Oxide		4 (33·3)	
NMBA		1 (8·3)	
ECMO		0	
Vasopressors		6 (50)	
Renal replacement therapy		3 (25)	
Diffuse alveolar damage on pathological examination, n (%)		9 (75)	
PaO2/FIO2 ratio (day of sample), mmHg		131 (104-155)	
Duration between MV initiation and tissue sampling, days		6 (2-13)	

ARDS: acute respiratory distress syndrome; RAS: renin-angiotensin system; APACHE II: Acute Physiology and Chronic Health Evaluation II; SOFA: Sequential Organ Failure Assessment; NMBA: Neuromuscular Blocking Agents; ECMO: Extra-Corporeal Membrane Oxygenation; LOS: Length of Stay; VFDays D28: Ventilatory-Free Days at Day 28; IQR: interquartile range.

Table S3. Reagents used for multiplex fluorescence immunohistochemistry.

Antigen	Primary antibody			Secondary antibody	Commonly associated cell types
	Dilution	company	Catalog number	Polymer HRP	
pIgR	1/5000	Santa Cruz	SC374343	Anti-mouse (Thermo B40961)	Airways ciliated cells
HIF-1α	1/500	Novus	NB100-449	Rabbit	Hypoxic cells
β-Tubulin IV	1/5000	Sigma	T7941	Anti-mouse	Airways ciliated cells
p63	1/1000	Dako	M731729	Anti-mouse	Airways basal cells
E-Cadherin	1/400	Dako	NCH-38	mouse	Epithelial cells
CA9	1/50	Bioké	#5649	Rabbit	Hypoxic cells
TGF-β2	1/20	R&D	DY302	Mouse	No specificity for any cell type

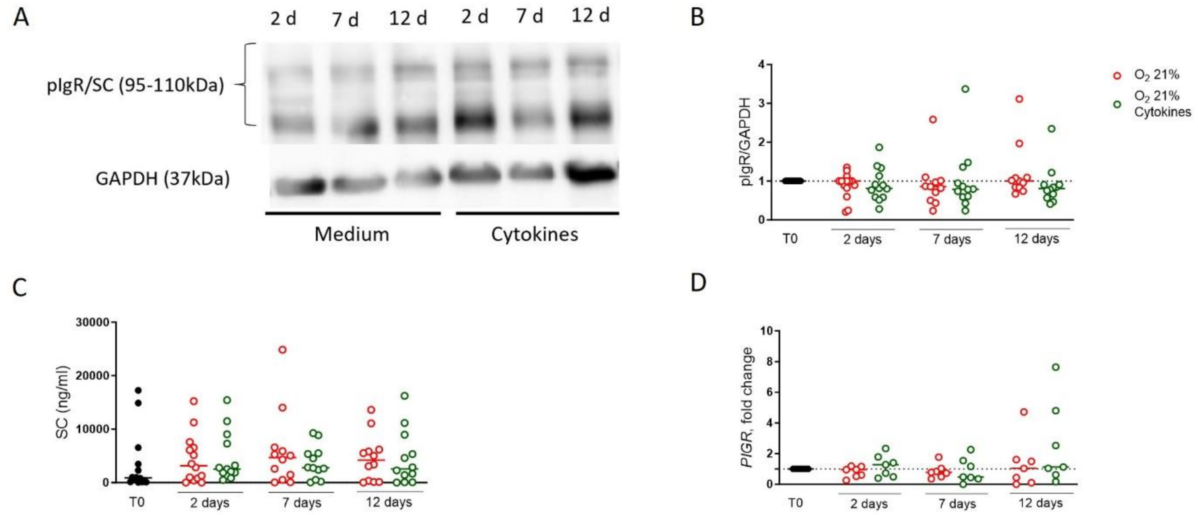

Figure S1. Inflammation effects on IgA/pIgR system.

A. Representative blot for pIgR and GAPDH in AE from one control grown under 21% O2 (left, "medium") or under 21% with 2.5 ng/ml of IL-1β, IL6 and TNF-α (right, "cytokines") for up to 12 days. B. Quantification of pIgR expression in ALI AE grown without (red) or with inflammatory cytokines (green) at baseline and after 2, 7 and 12 days, normalized for GAPDH (n=14). C. SC release in apical washes measured by ELISA (n=14) and quantification of PIGR mRNA expression, measured by RT-PCR and normalized for housekeeping genes (n=7) in the same conditions. Data are median ± IQR.

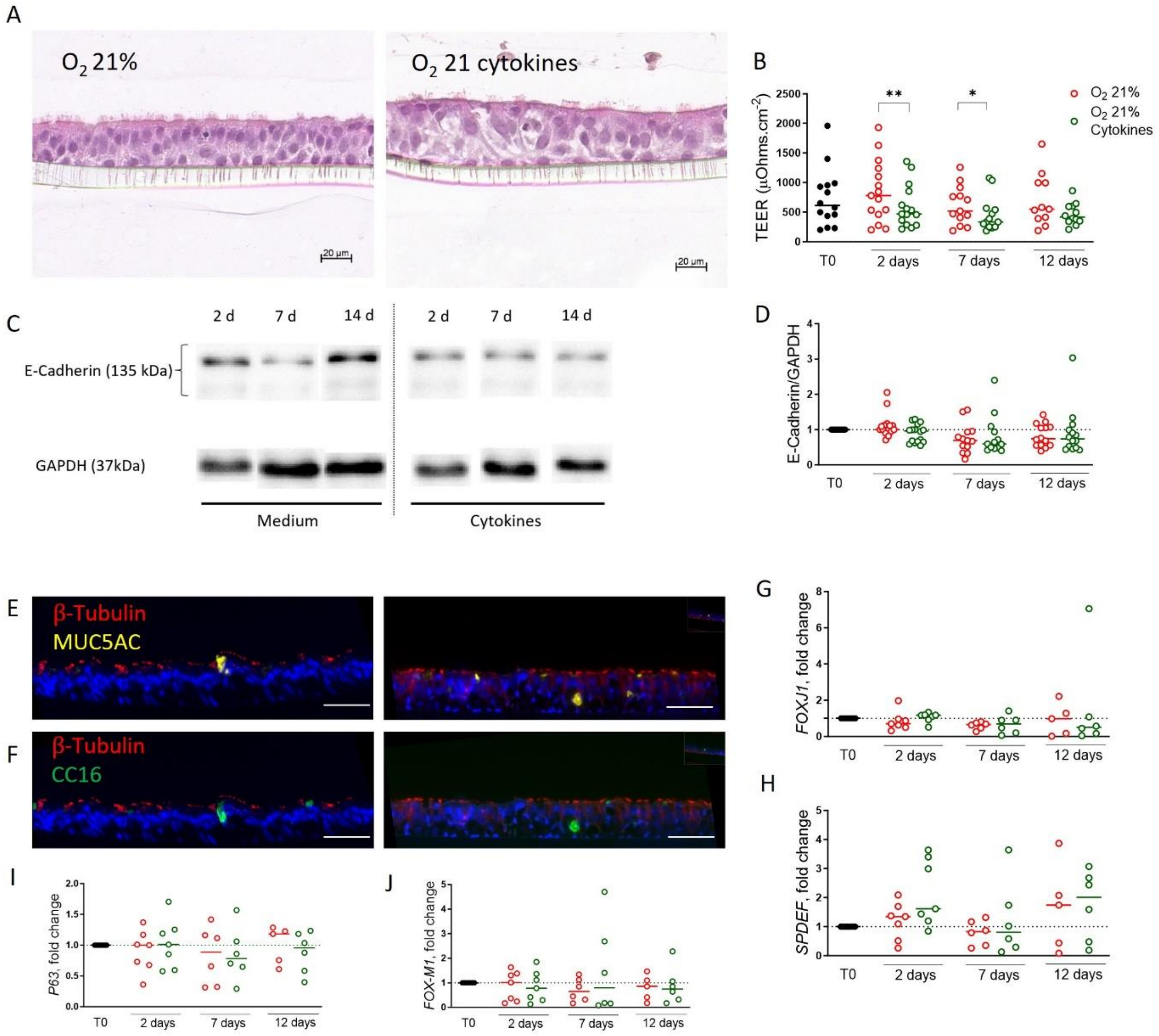

Figure S2. Inflammation effects on AE structure.

A. Representative Hematoxylin/Eosin-stained ALI AE from one control, grown for 12 days under 21% O2 without (left) or with inflammatory cytokines (right). B. Decreased Trans-Epithelial Electric Resistance (TER) in AE from controls (n=15) measured after 2 and 7 days in inflammatory conditions (green) compared to control conditions (red). C-D. Representative blot (C) and quantification (D) for E-cadherin and GAPDH in AE grown in control vs inflammatory conditions showing no change induced by inflammatory cytokines (n=14). E-F. Representative immunofluorescence staining of a control AE grown for 12 days under 21% O2 without (left) or with inflammatory cytokines (right), suggesting no change in the expression of β-tubulin, MUC5AC (E) or CC16 (F) in inflammatory conditions. Scale bars, 50µm. G-J. Quantification of *FOXJ1* (G), *SPDEF* (H), *P63* (I) and *FOX-M1* (J) mRNA measured by RT-qPCR and normalized for housekeeping genes (n=7). Data are median ± IQR. Between group difference was evaluated using a Wilcoxon test. *indicates $p<0{\cdot}05$, ** $p<0{\cdot}005$.
